# The legal implications of prenatal diagnosis in Malaysia

**DOI:** 10.12688/f1000research.73231.1

**Published:** 2021-11-01

**Authors:** Chee Ying Kuek

**Affiliations:** 1 Faculty of Law, Multimedia University, Malacca, 75450, Malaysia

**Keywords:** Abortion, disability, prenatal diagnosis, termination of pregnancy, wrongful birth, wrongful life

## Abstract

**Background**: Prenatal diagnosis enables detection of any disease or disability of the fetus during the pregnancy of a woman. Parents whose fetus is found to have a serious disorder from antenatal testing may terminate the pregnancy if it is permitted by the law or continue with the pregnancy to term. However, the chance of terminating a pregnancy may be denied if there is prenatal negligence by the medical practitioner in terms of diagnosis or failure to advise on the test results correctly. The purpose of this research is to examine the possible legal implications of prenatal diagnosis in Malaysia.

**Methods**: This study adopts doctrinal legal research in which the researcher examines statutes and decided cases in Malaysia, the United Kingdom (UK) and Singapore relating to abortion, wrongful birth and wrongful life claims, in order to determine the legal implications of prenatal diagnosis in Malaysia.

**Results**: In Malaysia, abortion following a prenatal diagnosis is only legally possible if the statutory criteria in the Penal Code are met. Abortion is illegal if it is not done for therapeutic purposes. A wrongful birth action brought by a woman who claims to be deprived of the opportunity to terminate her pregnancy may be successful in Malaysia, if it can be proven that a legal abortion could have been performed if not because of the prenatal negligence of the medical practitioner. However, a wrongful life action brought in the child’s name for being allowed to be born with a disability may not be viable since the claim could hardly be established and it is against the public policy.

**Conclusions**: Theoretically, it is possible to bring a wrongful birth action resulted from negligence in prenatal diagnosis successfully in Malaysia, but the chance is relatively slim for wrongful life action.

## Introduction

Prenatal diagnosis is offered to pregnant women with the aim of detecting any disease or disability of the fetus. The information about the serious genetic disorder or chromosomal abnormality detected in the fetus after the prenatal diagnosis may lead to the decision of terminating a pregnancy if it is permitted by the law. However, a prenatal diagnosis which is not properly conducted, or misinformation about the outcome of the prenatal tests, or a failure to advise a prospective mother about the serious medical condition of the fetus may result in the birth of a child with an undesirable disease, unexpected by the parents.

Previous studies have discussed the civil suits brought by parents or children against medical practitioners who were negligent in prenatal diagnosis, which led to the birth of children with severe disability or serious diseases, whom the parents would not have had if not because of the prenatal negligence. However, these studies mainly involved Western jurisdictions such as the United Kingdom (UK) ( Fordham, 2004; Todd, 2005, 2019; Mason, 2007), Australia ( Fordham, 2004; Todd, 2005, 2019), United States ( Donavan, 1984; Hermanson, 2019; Haqq, 2020) but rarely in Asian countries although there was discussion on the position in Singapore ( Fordham, 2005).

The purpose of this article is to examine the possible legal implications of prenatal diagnosis in Malaysia. Three aspects will be examined, namely the access to the abortion service, the possibility of wrongful birth and wrongful life claims in Malaysia. Discussion on these questions in the Asia region is relatively scarce especially in Malaysia due to the absence of reported judicial decisions in the country.

## Methods

This article adopts doctrinal legal research or “black-letter law” approach which involves identifying, analysing and synthesizing the content of the law. This method requires the examination of the essential features of the relevant statutes and the court judgments, followed by the synthesis of the elements to construct a statement of the law on the matter in question ( Hutchinson, 2013). Therefore, this study examined statutes, guidelines and decided cases in Malaysia, the UK and Singapore relating to abortion, wrongful birth and wrongful life claims. Searches for statutory provisions, cases and papers relating to abortion pursuant to prenatal diagnosis, wrongful birth and wrongful life cases were conducted in the databases and website namely LawNet, Westlaw Asia, Lexis Advance and Singapore Statute Online between 1 May 2021 and 14 July 2021. The search terms namely “abortion”, “termination of life”, “prenatal diagnosis”, “wrongful birth” and “wrongful life” were used during the searches in these databases and website.

Statutory provisions in the Penal Code (Malaysia), Abortion Act 1967 (UK), Infant Life (Preservation) Act 1929 (UK), Termination of Pregnancy Act (Chapter 324) (Singapore) and case law were identified and analysed to explore the possible legal liabilities of medical practitioners in Malaysia relating to this kind of prenatal negligence, as compared to their counterparts in the UK and Singapore. The statutory provisions were interpreted by giving their ordinary meaning unless it is inappropriate in the light of the context and purpose ( Sanson, 2016). The UK and Singapore were chosen for reference since these are common law countries which share the same legal system with Malaysia. In addition, there were reported cases of wrongful birth and wrongful life claims in these two jurisdictions. Four wrongful birth cases in the UK (2000-2001) and Singapore (2005) as well as two wrongful life cases in the UK (1982) and Singapore (2005) which were derived from the searches will be referred to in the discussion. These jurisdictions can offer experience and lessons to Malaysia since there is no reported case law in this aspect in Malaysia.

Ethical approval has been obtained from the Research Ethics Committee, Multimedia University (approval number: EA0482021).

## Results

### Abortion law in Malaysia

An induced abortion is generally prohibited under the Penal Code in Malaysia. Voluntarily causing a woman with child to miscarry constitutes a criminal offence. However, if it is performed by a registered medical practitioner who, in good faith, is of the view that the continuance of the pregnancy would risk the life of the pregnant woman or cause injury to her mental or physical health, greater than termination of pregnancy, then it is not an offence (Penal Code, s 312). An act with the intention to prevent the live birth of a child or cause the child to die after birth constitute an offence, unless it is done in good faith to save the mother’s life (Penal Code, s 315). From the provisions of the Penal Code, some observation can be made. Firstly, abortion is not available upon request and a woman does not have a legally enforceable right to abortion in Malaysia. Secondly, the legality of an abortion rests upon the medical practitioner’s views formed in good faith that continuance of pregnancy is posing greater risk than the termination of pregnancy. Thirdly, an abortion will be legal if the statutory criteria are met. Fourthly, a medical practitioner who performs an abortion will lose the legal protection if he is not acting in good faith. Therefore, a woman has no right to an abortion in Malaysia. However, if the medical practitioner decides that the women will suffer a greater risk of physical or mental health by having a child with serious fetal impairment and her health will be better served by termination of pregnancy, then termination of pregnancy will be lawful in such a circumstance. In Malaysia, termination of pregnancy is allowed until the 22 ^nd^ week of gestation or if the fetus is less than 500 grams ( Ministry of Health Malaysia, 2012).

### Abortion law in the United Kingdom and Singapore

In the UK, abortion is allowed if certain conditions are met. Two registered medical practitioners must form the opinion in good faith that any one of the four statutory grounds for abortion is satisfied: (a) the pregnancy shall not exceed 24 weeks of gestation and its continuance will cause a greater risk of injury to the physical or mental health of the woman or her existing child; (b) the abortion is needed to prevent grave permanent injury to the woman’s physical or mental health; (c) the continuance of the pregnancy would pose a greater risk to the woman’s life than the termination of pregnancy; (d) there is a substantial risk that the child to be born with physical or mental abnormalities (Abortion Act 1967, s 1(1)). It is also not an offence if a person causes the death of a child capable of being born alive, if it is done in good faith to preserve the mother’s life (Infant Life (Preservation) Act 1929, s 1(1)).

As for Singapore, termination of pregnancy is lawful if it is performed by an authorised medical practitioner upon the pregnant woman’s request and with her written consent (Termination of Pregnancy Act (Chapter 324), s 3(1)). However, abortion is not allowed if the pregnancy exceeds 24 weeks of gestation unless it is carried out “to save the life or to prevent grave permanent injury to the physical or mental health of the pregnant woman” (Termination of Pregnancy Act (Chapter 324), s 4(1)).

It would be observed that abortion is allowed on therapeutic and social grounds in the UK and Singapore. This is wider than the law in Malaysia which permits abortion on therapeutic reasons, to save the mother’s life or to avoid greater injury to her mental or physical health should the pregnancy be continued.

## Discussion

### Wrongful birth

A wrongful birth action may be brought on the basis that the negligent prenatal diagnosis, or misinformation about the outcome of the prenatal tests, or a failure to advise on the fetal impairment by the medical practitioner has led to the birth of a child with significant physical or mental abnormalities. Such prenatal negligence could have deprived the woman of the chance of making informed consent for a legal abortion. The claim is for damages associated with rearing the child with impairment, as the parents would have terminated the pregnancy but for the negligence of those charged with prenatal testing or diagnosis.

A plaintiff for a wrongful birth action must establish the existence of a duty of care, breach of duty and the causation of damage. Proving a duty of care should not pose any problem because a medical practitioner who performs a prenatal test and/or provides diagnostic and interpretive service for the prenatal test owes a duty of care to the patient. As for the breach of duty, a plaintiff needs to prove that the defendant has acted below the reasonable standard of care expected from a medical practitioner. Finally, a plaintiff must establish a clear chain of causation between the breach of the duty and the harm caused to the plaintiff.

In cases where the prenatal negligence led to the birth of an affected child, the courts in the UK allowed or recognised the recovery of costs for the child’s special needs or costs associated with the disability. However, the full costs of upbringing the child was denied. For instance, in the case of *Rand v East Dorset Health Authority* (2000), the antenatal test showed that the fetus was likely to have Down’s syndrome but the doctors had negligently omitted to inform the parents about it. It was held that the claimants could recover damages in respect of economic loss caused by the child’s disability. In *Hardman v Amin* (2001), a doctor failed to diagnose a rubella infection in a pregnant woman as he did not arrange seriological tests for her. The costs of providing for the disabled child’s special needs were held to be recoverable relating to the degree of disability, and it was not governed by the parents’ available resources. In the case of *Lee v Taunton and Somerset NHS Trust* (2001), the radiologist negligently failed to detect spina bifida in the fetus when the ultrasound scan was conducted. The court followed *Hardman* and recognised the parent’s right to recover the costs of meeting the disabled child’s special needs.

The High Court of Singapore had a chance to hear a wrongful birth and wrongful life case brought by a mother and an infant in *JU and Another v See Tho Kai Yin* (2005). The court dismissed the mother’s claims. Applying the test in *Bolam v Friern Hospital Management Committee* (1951), the court held that the medical practitioner had acted reasonably in managing the woman’s pregnancy in accordance with the practice since it was too late for the woman to undergo a legal abortion at the material time (24-25 weeks of gestation).

By reference to the cases in the UK and Singapore, it is theoretically possible for a woman to commence a wrongful birth action in Malaysia against the medical practitioner who commits prenatal negligence. However, the woman must be able to prove retrospectively that, a legal abortion could have been performed and if she had been informed or advised about the fetal abnormality, she would have terminated the pregnancy. Therefore, evidence which shows that the woman was very concerned with the possibility of fetal abnormality during her pregnancy would be helpful in supporting the claim. A woman who has an existing child affected by genetic disease or who has a history of mental health problem may prove through an expert witness that she is at risk of facing greater mental health problem due to the unwanted pregnancy of a prospective child with severe genetic disease. Nevertheless, wrongful birth action is expected to be rare in Malaysia due to the cultural and religious reasons which tend to be more pro-life than pro-choice ( Low, Tong, and Gunasegaran, 2013).

### Wrongful life

A wrongful life action is brought by or on behalf of a child born with disability or abnormalities in circumstances that if the medical practitioner’s negligence had not occurred, the child would not have been born at all. Such a claim may arise when the fetus’s abnormality was not detected by a prenatal test due to negligence.

In the leading English case of *McKay v Essex AHA* (1982), the wrongful life claim was dismissed. The court held that the doctor did not owe a legal duty under the Abortion Act 1967 to the fetus to terminate its life. The plaintiff’s claim was contrary to the public policy as it violated the sanctity of human life. Furthermore, it is impossible to measure appropriate damages. In the wrongful life claim in *JU and Another v See Tho Kai Yin* (2005), the High Court of Singapore adopted the common law position in the UK, Canada and Australia and rejected the claim.

If a wrongful life claim is commenced in Malaysia, it is very likely to fail as well. It would be against the public policy to rule that a disabled child should not have been born as it devalues the life of a disabled person and violates human dignity. It is also impossible for the disabled child to establish that he or she would have been better off not to have existed. The basic principle of tort compensation is to restore the plaintiff to the original state in the absence of the defendant’s negligence. However, in the case of wrongful life, there is difficulty in quantifying damages or compensation to the disabled child by comparing a life with serious disabilities with a state of non-existence.

### Limitation

This study sought to fill the gap of the prior academic work in which the legal implications of prenatal diagnosis were hardly discussed in the Asian context, particularly in Malaysia. However, the fact that there is no reported or decided case on wrongful birth and wrongful life in Malaysia has limited the study. This is because the analysis of the legal implications of prenatal diagnosis in Malaysia can only be based on the local legislation and guideline, as well as reference to the decided cases of other jurisdictions which have the persuasive value.

## Conclusions

Theoretically, a wrongful birth action brought by a woman who claims to be deprived of the opportunity to terminate her pregnancy may be successful in Malaysia. It needs to be proven that a legal abortion could have been performed and the woman would have chosen to terminate the pregnancy, if not because of the medical practitioner’s prenatal negligence. However, such action may be rare in Malaysia considering the cultural and religious reasons.

In contrast, a wrongful life action brought in the child’s name for being allowed to be born with a disability would most likely be rejected. This is because such action appears to undermine human life and it is against the public policy. In addition, the claim of being harmed by being born could hardly be established or recognised in law.

## Data availability

### Underlying data

Figshare: Legal implications of prenatal diagnosis. https://doi.org/10.6084/m9.figshare.15167907.v1 ( C Y Kuek, 2021).

This project contains the following underlying data.

• my Penal Code ss 312, 315.docx
• sg Ju and Another v See Tho Kai Yin (2005) SGHC 140.docx
• sg Termination of Pregnancy Act, s 3.docx
• uk Abortion Act 1967, s 1.rft
• uk Bolam v Friern Hospital Management Committee (1957) 1 WLR 582.rtf
• uk Hardman v Amin 59 BMLR 58.docx
• uk Infant Life (Preservation) Act 1929, s 1.rtf
• uk Lee v Taunton and Somerset NHS Trust (2001) 1 FLR 419.docx
• uk McKay and Another v Essex Area Health Authority.docx
• uk Rand v East Dorset Health Authority 56 BMLR 39.docx

Data is available under the terms of the Creative Commons Zero “No rights reserved” data waiver (CC0 1.0 Public domain dedication).
