# The legal implications of prenatal diagnosis in Malaysia

**DOI:** 10.12688/f1000research.73231.3

**Published:** 2022-09-16

**Authors:** Chee Ying Kuek

**Affiliations:** 1 Faculty of Law, Multimedia University, Malacca, 75450, Malaysia

**Keywords:** Abortion, disability, prenatal diagnosis, termination of pregnancy, wrongful birth, wrongful life

## Abstract

**Background**: Prenatal diagnosis enables detection of any disease or disability of the fetus during the pregnancy of a woman. Parents whose fetus is found to have a serious disorder from antenatal testing may terminate the pregnancy if it is permitted by the law or continue with the pregnancy to term. However, the chance of terminating a pregnancy may be denied if there is prenatal negligence by the medical practitioner in terms of diagnosis or failure to advise on the test results correctly. The purpose of this research is to examine the possible legal implications of prenatal diagnosis in Malaysia.

**Methods**: This study adopts doctrinal legal research in which the researcher examines statutes and decided cases in Malaysia, the United Kingdom (UK) and Singapore relating to abortion, wrongful birth and wrongful life claims, in order to determine the legal implications of prenatal diagnosis in Malaysia.

**Results**: In Malaysia, abortion following a prenatal diagnosis is only legally possible if the statutory criteria in the Penal Code are met. Abortion is illegal if it is not done for therapeutic purposes. A wrongful birth action brought by a woman who claims to be deprived of the opportunity to terminate her pregnancy may be successful in Malaysia, if it can be proven that a legal abortion could have been performed if not because of the prenatal negligence of the medical practitioner. However, a wrongful life action brought in the child’s name for being allowed to be born with a disability may not be viable since the claim could hardly be established and it is against the public policy.

**Conclusions**: Theoretically, it is possible to bring a wrongful birth action resulted from negligence in prenatal diagnosis successfully in Malaysia, but the chance is relatively slim for wrongful life action.

## Introduction

Prenatal diagnosis is offered to pregnant women with the aim of detecting any disease or disability of the fetus. The information about the serious genetic disorder or chromosomal abnormality detected in the fetus after the prenatal diagnosis may lead to the decision of terminating a pregnancy if it is permitted by the law. However, a prenatal diagnosis which is not properly conducted, or misinformation about the outcome of the prenatal tests, or a failure to advise a prospective mother about the serious medical condition of the fetus may result in the birth of a child with an undesirable disease, unexpected by the parents.

Previous studies have discussed the civil suits brought by parents or children against medical practitioners who were negligent in prenatal diagnosis, which led to the birth of children with severe disability or serious diseases, whom the parents would not have had if not because of the prenatal negligence. However, these studies mainly involved Western jurisdictions such as the United Kingdom (UK) ( Fordham, 2004; Todd, 2005, 2019; Mason, 2007), Australia ( Fordham, 2004; Todd, 2005, 2019), United States ( Donavan, 1984; Hermanson, 2019; Haqq, 2020) but rarely in Asian countries although there was discussion on the position in Singapore ( Fordham, 2005).

The purpose of this article is to examine the possible legal implications of prenatal diagnosis in Malaysia. However, the legal implications referred in this article will be considered specifically from viewpoints of abortion, the possibility of wrongful birth and wrongful life claims in Malaysia. Discussion on these questions in the Asia region is relatively scarce especially in Malaysia due to the absence of reported judicial decisions in the country.

## Methods

This article adopts doctrinal legal research or “black-letter law” approach which involves identifying, analysing and synthesizing the content of the law. This method requires the examination of the essential features of the relevant statutes and the court judgments, followed by the synthesis of the elements to construct a statement of the law on the matter in question ( Hutchinson, 2013). Therefore, this study examined statutes, guidelines and decided cases in Malaysia, the UK and Singapore relating to abortion, wrongful birth and wrongful life claims. Searches for statutory provisions, cases and papers relating to abortion pursuant to prenatal diagnosis, wrongful birth and wrongful life cases were conducted in the databases and website namely LawNet, Westlaw Asia, Lexis Advance and Singapore Statute Online between 1 May 2021 and 22 May 2022. The search terms namely “abortion”, “termination of life”, “prenatal diagnosis”, “wrongful birth” and “wrongful life” were used during the searches in these databases and website.

Statutory provisions in the Penal Code (Malaysia), Abortion Act 1967 (UK), Infant Life (Preservation) Act 1929 (UK), Congenital Disabilities (Civil Liability) Act 1976 (UK), Termination of Pregnancy Act (Chapter 324) (Singapore) and case law were identified and analysed to explore the possible legal liabilities of medical practitioners in Malaysia relating to this kind of prenatal negligence, as compared to their counterparts in the UK and Singapore. The statutory provisions were interpreted by giving their ordinary meaning unless it is inappropriate in the light of the context and purpose ( Sanson, 2016). The UK and Singapore were chosen for reference since these are common law countries which share the same legal system with Malaysia. In addition, there were reported cases of wrongful birth and wrongful life claims in these two jurisdictions. Four wrongful birth cases in the UK (2000-2001) and Singapore (2005) as well as three wrongful life cases in the UK (1982-2021) and Singapore (2005) which were derived from the searches will be referred to in the discussion. These jurisdictions can offer experience and lessons to Malaysia since there is no reported case law in this aspect in Malaysia.

Ethical approval has been obtained from the Research Ethics Committee, Multimedia University (approval number: EA0482021).

## Results

### Abortion law in Malaysia

An induced abortion is generally prohibited under the Penal Code in Malaysia. Voluntarily causing a woman with child to miscarry constitutes a criminal offence. However, if it is performed by a registered medical practitioner who, in good faith, is of the view that the continuance of the pregnancy would risk the life of the pregnant woman or cause injury to her mental or physical health, greater than termination of pregnancy, then it is not an offence (Penal Code, s 312). The term “abortion” is not found in the Penal Code. However, reference can be made to the Guideline on Termination of Pregnancy for Hospitals in the Ministry of Health, which defines it as “the expulsion or removal of an embryo or fetus from the uterus at a stage of pregnancy when it is incapable of independent survival” ( Ministry of Health Malaysia, 2012). The term “miscarriage” refers to the premature expulsion of the fetus from the mother’s womb at any period of the pregnancy before the term of gestation is completed ( Mallal, 2002). The court in Malaysia considered procuring a miscarriage as the same with procuring an abortion. This can be seen in the case of *PP v Dr Nadason Kanagalingam* (1985) where the accused, who was an obstetrician and gynaecologist, was convicted under s 312 of the Penal Code. The court used the terms “procuring the miscarriage” and “abortion” interchangeably. The accused was found guilty of the offence of causing miscarriage as he performed the abortion when there was no indication that the woman’s life was endangered if the pregnancy were to continue.

An act with the intention to prevent the live birth of a child or cause the child to die after birth constitute an offence, unless it is done in good faith to save the mother’s life (Penal Code, s 315). From the provisions of the Penal Code, some observation can be made. Firstly, abortion is not available upon request and a woman does not have a legally enforceable right to abortion in Malaysia. Secondly, the legality of an abortion rests upon the medical practitioner’s views formed in good faith that continuance of pregnancy is posing greater risk than the termination of pregnancy. Thirdly, an abortion will be legal if the statutory criteria are met. Fourthly, a medical practitioner who performs an abortion will lose the legal protection if he is not acting in good faith. Therefore, a woman has no right to an abortion in Malaysia. However, if the medical practitioner decides that the women will suffer a greater risk of physical or mental health by having a child with serious fetal impairment and her health will be better served by termination of pregnancy, then termination of pregnancy will be lawful in such a circumstance. It is not necessary for the attending doctor to get the opinion from a psychologist or psychiatrist for the mental health assessment. In Malaysia, termination of pregnancy is allowed until the 22 ^nd^ week of gestation or if the fetus is less than 500 grams ( Ministry of Health Malaysia, 2012).

### Abortion law in the United Kingdom and Singapore

In the UK, abortion is allowed if certain conditions are met. Two registered medical practitioners must form the opinion in good faith that any one of the four statutory grounds for abortion is satisfied: (a) the pregnancy shall not exceed 24 weeks of gestation and its continuance will cause a greater risk of injury to the physical or mental health of the woman or her existing child; (b) the abortion is needed to prevent grave permanent injury to the woman’s physical or mental health; (c) the continuance of the pregnancy would pose a greater risk to the woman’s life than the termination of pregnancy; (d) there is a substantial risk that the child to be born with physical or mental abnormalities ( Abortion Act 1967, s 1(1)). In determining the first ground, namely whether there is any risk of injury to the woman or her existing children’s physical or mental health, the registered medical practitioners may take into account the pregnant woman’s “actual or reasonably foreseeable environment” ( Abortion Act 1967, s 1(2)). Commentators have referred this as “social ground” as it may encompass social consideration of inconvenience and social pressure ( Pattinson, 2020). It is also not an offence if a person causes the death of a child capable of being born alive, if it is done in good faith to preserve the mother’s life ( Infant Life (Preservation) Act 1929, s 1(1)).

As for Singapore, termination of pregnancy is lawful if it is performed by an authorised medical practitioner upon the pregnant woman’s request and with her written consent ( Termination of Pregnancy Act (Chapter 324), s 3(1)). However, abortion is not allowed if the pregnancy exceeds 24 weeks of gestation unless it is carried out “to save the life or to prevent grave permanent injury to the physical or mental health of the pregnant woman” ( Termination of Pregnancy Act (Chapter 324), s 4(1)).

It would be observed that abortion is allowed on therapeutic and social grounds in the UK and Singapore. This is wider than the law in Malaysia which only permits abortion on therapeutic reasons, namely to save the mother’s life or to avoid greater injury to her mental or physical health should the pregnancy be continued. Social consideration such as social pressure faced by the pregnant woman cannot be taken into account by the registered medical practitioners in Malaysia, if there is no indication that the risk of continuing the pregnancy is greater than the risk of terminating the pregnancy. Furthermore pregnancy of not more than 24 weeks duration may be terminated in Singapore upon the woman’s request and consent. Such an abortion is legal in Singapore and the medical practitioner is not required to find the continuance of pregnancy is posing a greater risk than termination of pregnancy before he performs the abortion. This is different from Malaysia where abortion cannot be performed upon a pregnant woman’s request, but the medical practitioner is the one who assesses the medical circumstances and decides if an abortion should be carried out. It is also observed that abortion is allowed in the UK if there is a high risk that the child will be born with physical or mental disabilities, based on the result of the prenatal diagnosis. However, it will not be legal to terminate a pregnancy in Malaysia solely on the ground of serious fetal impairment, discovered by prenatal diagnosis. An abortion can only be legally performed if the medical practitioner finds that the continuance of pregnancy in which the fetus is affected by serious abnormalities will pose a greater risk to the prospective mother’s physical or mental health.

## Discussion

### Wrongful birth

A claim on medical negligence, which applies the principles of tortious claims, must establish the existence of a duty of care, breach of the duty and the causation of damage. A medical practitioner owes a duty to take reasonable care towards the patient. Breach of duty arises when the medical practitioner falls below the reasonable standard of care required of him. To succeed in the medical negligence claim, there must be a clear chain of causation between the breach of duty and the claimant’s injury, loss or damage.

According to the test in *Bolam v Friern Hospital Management Committee* (1951) or what is commonly known as the *Bolam* test, a medical practitioner is required to exercise reasonable care and skill expected of an ordinary competent medical practitioner. He is not negligent if he has acted in accordance with a practice accepted as proper by a responsible body of medical opinion. The doctor-centric *Bolam* test was revisited in the case of *Bolitho v City and Hackney Health Authority* (1997), in which the House of Lords held that a medical practitioner is not negligent in respect of diagnosis and treatment if the body of medical opinion relied upon has a logical basis, or is responsible, reasonable or respectable. In the subsequently case of *Montgomery v Lanarkshire Health Board* (2015), the Supreme Court did not apply *Bolam* test in respect of provision of advice and disclosure of risk. It was held that a medical practitioner has a duty to disclose to the patient about the material risks of the proposed treatment. The materiality of risk is tested against what a reasonable patient would attach significance to. This patient-centric position is similar to the Australian case of *Rogers v Whitake* (1992), in which a doctor is under a duty to advise a patient on a material risk in the proposed treatment.

In Malaysia, the Federal Court in the cases of *Zulhasnimar bt Hasan Basri & Anor v Dr Kuppu Velumani P & Ors* (2017) and *Dr Hari Krishnan v Megat Noor Ishak bin Megat Ibrahim & Anor and another Appeal* (2018) made a distinction between the standard of care in respect of diagnosis and treatment in which the *Bolam* test applies; and the standard of care in respect of duty to advise of risk in which the “prudent patient” test applies.

A wrongful birth action refers to a claim made by parents who have been deprived of the opportunity to avoid the continuance of an existing pregnancy. It may be brought on the basis that the negligent prenatal diagnosis, or misinformation about the outcome of the prenatal tests, or a failure to advise on the fetal impairment by the medical practitioner has led to the birth of a child with significant physical or mental abnormalities. Such prenatal negligence could have deprived the woman of the chance of making informed consent for a legal abortion. The claim is for damages associated with rearing the child with impairment, as the parents would have terminated the pregnancy but for the negligence of those charged with prenatal testing or diagnosis.

A plaintiff for a wrongful birth action must establish the existence of a duty of care, breach of duty and the damage was caused by the breach of duty. Proving a duty of care should not pose much problem because a medical practitioner who performs a prenatal test and/or provides diagnostic and interpretive service for the prenatal test owes a duty of care to the patient. As for the breach of duty, a plaintiff needs to prove that the defendant has acted below the reasonable standard of care expected from a medical practitioner. Finally, a plaintiff must establish a clear chain of causation between the breach of the duty and the harm caused to the plaintiff.

In cases where the prenatal negligence led to the birth of an affected child, the courts in the UK allowed or recognised the recovery of costs for the child’s special needs or costs associated with the disability. However, the full costs of upbringing the child was denied. For instance, in the case of *Rand v East Dorset Health Authority* (2000), the antenatal test showed that the fetus was likely to have Down’s syndrome but the doctors had negligently omitted to inform the parents about it. It was held that the claimants could recover damages in respect of economic loss caused by the child’s disability. In *Hardman v Amin* (2001), a doctor failed to diagnose a rubella infection in a pregnant woman as he did not arrange seriological tests for her. The costs of providing for the disabled child’s special needs were held to be recoverable relating to the degree of disability, and it was not governed by the parents’ available resources. In the case of *Lee v Taunton and Somerset NHS Trust* (2001), the radiologist negligently failed to detect spina bifida in the fetus when the ultrasound scan was conducted. The court followed *Hardman* and recognised the parent’s right to recover the costs of meeting the disabled child’s special needs.

The High Court of Singapore had a chance to hear a wrongful birth and wrongful life case brought by a mother and an infant in *JU and Another v See Tho Kai Yin* (2005). The court dismissed the mother’s claims. Applying the *Bolam* test, the court held that the medical practitioner had acted reasonably in managing the woman’s pregnancy in accordance with the practice since it was too late for the woman to undergo a legal abortion at the material time (24-25 weeks of gestation).

By reference to the cases in the UK and Singapore, it is theoretically possible for a woman to commence a wrongful birth action in Malaysia against the medical practitioner who commits prenatal negligence. Depending on the nature of the prenatal negligence, the *Bolam* test will apply to negligent diagnosis and treatment while the prudent patient test in *Montgomery* will apply if there is a failure or omission to advise the patient on material risk, which results in the birth of an affected child. However, the woman must be able to prove retrospectively that, a legal abortion could have been performed and if she had been informed or advised about the fetal abnormality, she would have terminated the pregnancy. Therefore, evidence which shows that the woman was very concerned with the possibility of fetal abnormality or attached significance to such risk during her pregnancy would be helpful in supporting the claim.

For instance, a woman who has an existing child affected by genetic disease or who has a history of mental health problem may prove through an expert witness that she is at risk of facing greater mental health problem due to the unwanted pregnancy of a prospective child with severe genetic disease. Likewise, if a child was born with serious impairment which has caused grave permanent injury to the woman’s mental health, it may give rise to a wrongful birth action. This is because the injury to her mental health could have been avoided, if the doctor was not negligent during the prenatal diagnosis or had advised her about the fetal impairment detected through the prenatal diagnosis. Nevertheless, wrongful birth action is expected to be rare in Malaysia due to the cultural and religious reasons which tend to be more pro-life than pro-choice ( Low, Tong, and Gunasegaran, 2013). In a study conducted on 116 parents in Malaysia who have children affected by thalassaemia, 83 parents (71.6%) were in favour of prenatal diagnosis and from these 83, only 33 (39.8%) supported termination of pregnancy that carries a fetus affected by thalassaemia major. In fact, 77.6% of the respondents who disapproved abortion cited their religion as the main reason of disapproval ( Ngim *et al*., 2013). In addition, some of the medical practitioners may be affected by the social-cultural norm which treats abortion as immoral ( Lim *et al*., 2020). Abortion is considered taboo in Malaysia and a party who brings a wrongful birth action may face social condemnation or moral opposition. The aggrieved party may also find it difficult to get an expert witness to support her wrongful birth claim.

### Wrongful life

A wrongful life action is brought by or on behalf of a child born with disability or abnormalities in circumstances that if the medical practitioner’s negligence had not occurred, the child would not have been born at all. Such a claim may arise when the fetus’ abnormality was not detected by a prenatal test due to negligence. Since 1976, prenatal injuries have been covered by the Congenital Disabilities (Civil Liability) Act 1976 (“the 1976 Act”). Under the 1976 Act, a child who is born with impairment caused by an occurrence before its birth can claim against a negligent defendant (other than the mother) who is answerable for the occurrence. The occurrence must be one which affected either parent’s ability to have a healthy child (pre-conception negligence), or injuries sustained during pregnancy or childbirth (in utero or post-conception negligence). The liability under the 1976 Act also extends to the negligent selection of embryos in an assisted reproductive treatment which results in the birth of a disabled child ( Congenital Disabilities (Civil Liability) Act 1976, ss 1(1), 1(2), 1A(1)(b) and (c)). Commentators found that the 1976 Act has inadvertently allowed statutory action for wrongful life in the context of assisted reproduction ( Scott 2013; Jackson 2016).

In the leading English case of *McKay v Essex AHA* (1982), the second plaintiff (mother) contracted rubella (German measles) in her early months of pregnancy. As a result, the first plaintiff (child) was born partly blind and deaf. The plaintiffs contended that the mother would have had an abortion if she had been informed that she had contracted rubella and advised of the risks to her child. The wrongful life claim was dismissed. The court held that the doctor did not owe a legal duty under the Abortion Act 1967 to the fetus to terminate its life. The plaintiff’s claim was contrary to the public policy as it violated the sanctity of human life. Furthermore, it is impossible to measure appropriate damages for the difference between the child’s impaired state and non-existence. It should be noted that the child in McKay case was born before the coming into force of the 1976 Act.

However, in the recent case of *Toombes v Mitchell* (2021), a wrongful life claim was successfully made by the claimant against her mother’s general practitioner, Dr Philip Mitchell. The claimant was born with a neural tube defect. She claimed that had the doctor given proper advice to her mother about the role of folic acid supplements in preventing spina bifida and neural tube defects, the mother would have delayed conception. The claimant would not have been conceived and born with the disability. Lambert J distinguished McKay case and held that the liability under s 1 of the 1976 Act was established as there was negligent advice leading to the claimant’s mother’s pregnancy despite her deficiency of folic acid, which resulted in the birth of the claimant with disabilities ( *Toombes v Mitchell*, 2020). Subsequently, HHJ COE QC ruled in favour of the claimant on the ground that her mother was not advised according to the guideline to take folic acid. Had she known that folic acid may prevent spinal bifida, she would have deferred her conception and given birth to a normal healthy child ( *Toombes v Mitchell,* 2021).

In the wrongful life claim in *JU and Another v See Tho Kai Yin* (2005), the High Court of Singapore adopted the common law position in the UK, Canada and Australia and rejected the claim. It was held that the doctor did not owe any duty to the fetus to advise the mother to terminate the pregnancy or to end the life of the fetus. Furthermore, even if there was any prenatal negligence on the part of the doctor, it did not result in the child’s Down’s syndrome since the disease was genetic in nature.

If a wrongful life claim is commenced in Malaysia, it is very likely to fail as well. Unlike the United Kingdom which has the 1976 Act, Malaysia does not have any statute which provides for cause of action for prenatal injuries. Therefore, any wrongful life claim will be brought as a common law action. It would be against the public policy to rule that a disabled child should not have been born as it undermines the sanctity of life, devalues the life of a disabled person and violates human dignity. The court would also be reluctant to allow such claim as it seems to have eugenic implications. It is also impossible for the disabled child to establish that he or she would have been better off not to have existed. The basic principle of tort compensation is to restore the plaintiff to the original state in the absence of the defendant’s negligence. However, in the case of wrongful life, there is difficulty in quantifying damages or compensation to the disabled child by comparing a life with serious disabilities with a state of non-existence.

### Limitation

This study sought to fill the gap of the prior academic work in which the legal implications of prenatal diagnosis from the viewpoints of abortion, the possibility of wrongful birth and wrongful life claims were hardly discussed in the Asian context, particularly in Malaysia. However, the fact that there is no reported or decided case on wrongful birth and wrongful life in Malaysia has limited the study. This is because the analysis of the legal implications of prenatal diagnosis in Malaysia can only be based on the local legislation and guideline, as well as reference to the decided cases of other jurisdictions which have the persuasive value.

## Conclusions

Theoretically, a wrongful birth action brought by a woman who claims to be deprived of the opportunity to terminate her pregnancy may be successful in Malaysia. It needs to be proven that a legal abortion could have been performed and the woman would have chosen to terminate the pregnancy due to the grave injury caused to her mental health, if not because of the medical practitioner’s negligence in the prenatal diagnosis, or failure to advise about the serious fetal impairment detected during the prenatal diagnosis. However, such action may be rare in Malaysia considering the cultural and religious reasons which tend to perceive abortion as immoral, and there is social stigma associated with abortion. It may also be challenging to get an expert witness to testify in the court for a wrongful birth action in Malaysia based on the same ground.

In contrast, a wrongful life action brought in the child’s name for being allowed to be born with a disability would most likely be rejected in Malaysia in the absence of a specific statute on prenatal injuries. This is because such action appears to undermine human life and it is against the public policy to rule that the child with disability should not have been born. In addition, comparing an impaired life and non-existence for the purpose of assessing damages is impossible since the latter cannot be known. The claim of being harmed by being born could hardly be established or recognised in law.

## Data availability

### Underlying data

Figshare: Legal implications of prenatal diagnosis. https://doi.org/10.6084/m9.figshare.15167907.v5 ( C Y Kuek, 2022).

This project contains the following underlying data.

• my Penal Code ss 312, 315.docx
• my *Dr Hari Krishnan v Megat Noor Ishak bin Megat Ibrahim & Anor and another Appeal*: 2018. 3 MLJ 281.docx
• my Public Prosecutor v Dr Nadason Kanagalingam [1985] 2 MLJ 122.docx
• my Zulhasnimar bt Hasan Basri & Anor v Dr Kuppu Velumani P & Ors [2017] 5 MLJ 438.docx
• sg Ju and Another v See Tho Kai Yin (2005) SGHC 140.docx
• sg Termination of Pregnancy Act, s 3.docx
• uk Abortion Act 1967, s 1.rft
• uk Bolam v Friern Hospital Management Committee (1957) 1 WLR 582.rtf
• uk Bolitho v City and Hackney Health Authority (1997) 4 All ER 771.docx
• uk Congenital Disabilities (Civil Liability) Act 1976.docx
• uk Hardman v Amin 59 BMLR 58.docx
• uk Infant Life (Preservation) Act 1929, s 1.rtf
• uk Lee v Taunton and Somerset NHS Trust (2001) 1 FLR 419.docx
• uk McKay and Another v Essex Area Health Authority.docx
• uk Rand v East Dorset Health Authority 56 BMLR 39.docx
• uk Toombes v Mitchell [2020] EWHC 3506.
• uk Toombes v Mitchell [2021] EWHC 3234.

Data is available under the terms of the Creative Commons Zero “No rights reserved” data waiver (CC0 1.0 Public domain dedication).
