# Supplementary material for: The legal implications of prenatal diagnosis in Malaysia
Source: F1000Res. 2022 Sep 16;10:1103. Originally published 2021 Nov 1. [Version 3] doi: 10.12688/f1000research.73231.3 (PMC9445564; doi:10.12688/f1000research.73231.3)
Supplement: Peer review history

# Peer review: Reviewer response for version 2

**DOI:** 10.5256/f1000research.135620.r147810

**Published:** 2022-09-05

**Authors:** Noraiza Abdul Rahman

**Affiliations:** 1 Faculty of Law, Universiti Teknologi MARA, Shah Alam, Malaysia

This work is an exciting study on the legal implications of prenatal diagnosis in Malaysia, and the author has provided a unique discussion on that particular issue. The work is generally well-written and structured. However, in my opinion, this work has some shortcomings regarding some data analyses and text, and I feel this unique analysis and discussion has not been utilised to its full extent. In several instances, I do think that the main discussion on the laws about abortion in Malaysia as well as wrongful birth is not supported by solid arguments. The arguments would be much more persuasive if the current literature provided had been fully elaborated and criticised the primary issue. Given these shortcomings, the manuscript only requires minor revisions.

 Specific comments:

- Abortion in Malaysia is governed under Sections 312 to 316 of the Penal Code. Do you have any reason not to mention or relate to other sections?
- *You stated, "* *Abortion is considered taboo in Malaysia and a party who brings a wrongful birth action may face social condemnation or moral opposition”. *Do you have any evidence or data to support this?
- You stated that the High Court of Singapore had a chance to hear a wrongful birth and wrongful life case brought by a mother and an infant in *JU and Another v See Tho Kai Yin (2005). *What do you think of the recent judgment in the case of *ACB v Thomson Medical Pte Ltd [2015] r*egarding a claim to recover the cost of raising a healthy baby born following a negligently performed IVF procedure? Should you include this in your arguments?

Is the work clearly and accurately presented and does it cite the current literature?

Yes

If applicable, is the statistical analysis and its interpretation appropriate?

Not applicable

Are all the source data underlying the results available to ensure full reproducibility?

Yes

Is the study design appropriate and is the work technically sound?

Yes

Are the conclusions drawn adequately supported by the results?

Yes

Are sufficient details of methods and analysis provided to allow replication by others?

Yes

Reviewer Expertise:

Medical Law, Law of Torts, Remedies in Torts

I confirm that I have read this submission and believe that I have an appropriate level of expertise to confirm that it is of an acceptable scientific standard.

# Peer review: Reviewer response for version 2

**DOI:** 10.5256/f1000research.135620.r147708

**Published:** 2022-08-25

**Authors:** Tengku Noor Azira Tengku Zainudin, Mohd. Zamre bin Mohd. Zahir

**Affiliations:** 1 Faculty of Law, Universiti Kebangsaan Malaysia, Selanggor, Malaysia

A. Comments by reviewer: Associate Prof. Dr. Tengku Noor Azira bt Tengku Zainudin:

1. It is suggested that the author provides a brief definition of abortion and whether it differs from the term miscarriage which is the term used in section 312 of the Malaysian Penal Code.
2. Discuss 1 or 2 relevant cases on section 312 (especially on the exceptions). This is to ensure that the readers will have a better understanding of the application of the section.
3. It was stated by the author that; "A woman who has an existing child affected by genetic disease or who has a history of mental health problems may prove through an expert witness that she is at risk of facing greater mental health problems due to the unwanted pregnancy of a prospective child with severe genetic disease." What about women who have no existing child affected by genetic disease and women who, at the time of the pregnancy, are not suffering from any mental health disorders? Is there a possibility that they can also use the exception under section 312, claiming that if the pregnancy is not terminated, it will affect their mental health?(Meaning the pregnancy will cause grave permanent injury to the woman’s mental health).
4. It would be better if the author could analyse section 312 to determine whether it is legally allowed to terminate the pregnancy solely on fetus factor, i.e., the abortion is done due to abnormalities of the unborn fetus, discovered through prenatal tests. This will enable the readers to see the difference between the Malaysian legal position and section 1(1)(d) Abortion Act 1967 in the UK regarding abortion due to the possibility that the fetus is suffering from physical or mental abnormalities, based on the result of prenatal diagnostic tests.
5. The author submitted that; "wrongful birth action is expected to be rare in Malaysia due to the cultural and religious reasons which tend to be more pro-life than pro-choice (Low, Tong, and Gunasegaran, 2013). Abortion is considered taboo in Malaysia and a party who brings a wrongful birth action may face social condemnation or moral opposition." It is in my opinion unnecessary to make this statement if there is no further explanation or discussion being made to support or justify the statement.

B. Comments by co-reviewer: Dr. Mohd. Zamre bin Mohd. Zahir:

1. The paper has been written properly and in a good manner. However, there are some improvements that need to be made accordingly.
2. Please re-write and rephrase the conclusion part. The previous conclusion is: Theoretically, it is possible to bring a wrongful birth action resulting from negligence in prenatal diagnosis successfully in Malaysia, but the chance is relatively slim for wrongful life action. (Please re-write and rephrase the conclusion part). The conclusion part seems very general and did not cover the crux of the paper.
3. When discussing Penal Code (Malaysia), kindly elaborate on the relevant sections as stated in the statute. The author did mention sections 312 and 315 of the Penal Code, but it is important to link the relevant sections with his/her current paper/study. Further, sections 316 and 318 are also applicable, if possible, to be discussed.
4. The author mentioned about this “In contrast, a wrongful life action brought in the child’s name for being allowed to be born with a disability would most likely be rejected. This is because such action appears to undermine human life and it is against public policy.” Expand this part with relevant justifications and arguments and please refer to the cases discussed earlier. 

Is the work clearly and accurately presented and does it cite the current literature?

Yes

If applicable, is the statistical analysis and its interpretation appropriate?

Not applicable

Are all the source data underlying the results available to ensure full reproducibility?

Yes

Is the study design appropriate and is the work technically sound?

Yes

Are the conclusions drawn adequately supported by the results?

Partly

Are sufficient details of methods and analysis provided to allow replication by others?

Yes

Reviewer Expertise:

Tort Law, Medical Law (Consent to treatment, Medical negligence)

We confirm that we have read this submission and believe that we have an appropriate level of expertise to confirm that it is of an acceptable scientific standard, however we have significant reservations, as outlined above.

# Peer review: Reviewer response for version 2

**DOI:** 10.5256/f1000research.135620.r142921

**Published:** 2022-07-01

**Authors:** Pin Lean Lau

**Affiliations:** 1 Brunel Law School, Brunel University, Uxbridge, UK; 2 Centre for Artificial Intelligence: Social & Digital Innovations, Brunel University London, London, UK

I have no further comments to make. Thank you for kindly taking into consideration my comments.

Is the work clearly and accurately presented and does it cite the current literature?

Partly

If applicable, is the statistical analysis and its interpretation appropriate?

Not applicable

Are all the source data underlying the results available to ensure full reproducibility?

No source data required

Is the study design appropriate and is the work technically sound?

Yes

Are the conclusions drawn adequately supported by the results?

Partly

Are sufficient details of methods and analysis provided to allow replication by others?

Yes

Reviewer Expertise:

European, international, and comparative law for genome editing (with a focus on pre-implantation genetic diagnosis, reproductive technologies and women's bodies; and the proliferation of virulent gene-edited pathogens and global bio-security); propertization and commodification studies of genetic materials and biomedical technologies; and the ethico-legal governance for artificial intelligence (AI) systems (with a focus on protection of fundamental rights, spatial 'body citizenship' and bio-constitutional implications of the AI-augmented biological human body.

I confirm that I have read this submission and believe that I have an appropriate level of expertise to confirm that it is of an acceptable scientific standard.

# Peer review: Reviewer response for version 1

**DOI:** 10.5256/f1000research.76871.r99451

**Published:** 2021-12-03

**Authors:** Pin Lean Lau

**Affiliations:** 1 Brunel Law School, Brunel University, Uxbridge, UK; 2 Centre for Artificial Intelligence: Social & Digital Innovations, Brunel University London, London, UK

Specific comments and suggestions to the author have been provided in a copy of the document, highlighted in yellow. Please also refer to the copy of the document which highlights these comments and suggestions, in addition to the general feedback below:

1. For example, in the introduction, the phraseology "legal implications of prenatal diagnosis" is much broader than the trajectory of the article suggests. As such, I have suggested that disclaimers or a narrow conception be explained, that the legal implications are considered specifically from the viewpoint of abortions, wrongly life, and wrongful birth claims. 
2. In the final paragraph of the section addressing abortion in the UK and Singapore, it would be helpful for the author to provide more clarity as to what "social grounds" would encompass, and specifically how this differs from the situation in Malaysia. The recommendation is not to use "social grounds" as it seems to denote a form of casualness - instead, it is more helpful to specify what these "social grounds" are, and to contrast this against Malaysia. 
3. Under the Discussion section on Wrongful Birth, it would provide greater clarity for the author to explain that the duty of care of a medical professional arises under the tort of negligence in common law jurisdictions. On the assumption that this article may be read beyond the scope of Malaysia and its neighbours, this would provide clarity for readers from civil law jurisdictions that do not strictly speaking, encounter medical negligence as a tortious cause of action. 
4. Under the same section, it would be good to highlight the Bolam and Bolitho cases in the UK, as these would be critical starting points of discussion of medical negligence in the UK, before theorizing on the wrongful birth claims. It would also be appropriate to reflect on Montgomery v Lanarkshire Health Board in the UK, as this case seems to suggest a displacement of the Bolam test in the UK (although it does not expressly overrule the Bolam case). 
5. The same strand of argument may be applied in the consideration of Malaysia's situation - i.e. Foo Fio Na v Dr Soo Fook Mun which also displaces the Bolam test - and contrasted this against the Singaporean position which continues to affirm the goodness of the Bolam principles. These would add a deeper dimension to the article in terms of reflecting the struggles and applicability even in the UK and Singapore. I also highlighted that even before considering wrongful birth or life in Malaysia, it is worth looking at medical negligence as an umbrella overview- and determining the possibility of wrongful life and birth claims being sought under this heading. 
6. The Discussions on Wrongful Life seem to be a little sparse in comparison with Wrongful Birth. I would suggest expanding on McKay v Essex and JU and Another in allowing a more nuanced discussion of wrongful life claims. It is also vital to discuss Evie Toombes v Dr Philip Mitchell (2020) as this case provides to us a renewed understanding as to how courts may consider wrongful life cases. In fact, Evie Toombes is distinguished from McKay, although its real outcome was based on the issue of causation. I would highly recommend that Evie Toombes be included in the narrative too. 

Is the work clearly and accurately presented and does it cite the current literature?

Partly

If applicable, is the statistical analysis and its interpretation appropriate?

Not applicable

Are all the source data underlying the results available to ensure full reproducibility?

No source data required

Is the study design appropriate and is the work technically sound?

Yes

Are the conclusions drawn adequately supported by the results?

Partly

Are sufficient details of methods and analysis provided to allow replication by others?

Yes

Reviewer Expertise:

European, international, and comparative law for genome editing (with a focus on pre-implantation genetic diagnosis, reproductive technologies and women's bodies; and the proliferation of virulent gene-edited pathogens and global bio-security); propertization and commodification studies of genetic materials and biomedical technologies; and the ethico-legal governance for artificial intelligence (AI) systems (with a focus on protection of fundamental rights, spatial 'body citizenship' and bio-constitutional implications of the AI-augmented biological human body.

I confirm that I have read this submission and believe that I have an appropriate level of expertise to confirm that it is of an acceptable scientific standard, however I have significant reservations, as outlined above.

# Response

**Published:** 2022-06-27

**Authors:** Chee Ying Kuek

**Affiliations:** Faculty of Law, Multimedia University, Malaysia

The author would like to thank Dr Pin Lean Lau for her helpful comments.

 This revision (version 2) addressed the comments and suggestions raised by Dr Pin Lean Lau. Amendments were made to the Introduction by adding the disclaimer as suggested. Minor amendments were made to the Methods by adding one statute and one new case, in which the judgment of the case was not available at the time of initial writing. Under the section “Abortion law in the United Kingdom and Singapore”, explanation was added on what was meant by “social ground” as referred by commentators. Under the Discussion section on “Wrongful Birth”, explanation was added in respect of a medical professional’s duty of care under the tort of medical negligence, the standard of care for diagnosis and treatment under the *Bolam* test which was subsequently revisited by *Bolitho*; and the “prudent patient” test for duty to advise in *Montgomery* case. The current position of Malaysia in respect of the standard of care was briefly pointed out through the Federal Court cases of *Zulhasnimar* and *Dr Hari Krishnan*. As for the Discussion section for “Wrongful Life”, the discussion of the Congenital Disabilities (Civil Liability) Act 1976 (the 1976 Act) and the new case of *Toombes v Mitchell* have been added. However, it was made clearer that Malaysia is still likely to adopt the common law position since Malaysia does not have similar statute like the 1976 Act.
