# Supplementary material for: The legal implications of prenatal diagnosis in Malaysia
Source: F1000Res. 2022 Jul 1;10:1103. Originally published 2021 Nov 1. [Version 2] doi: 10.12688/f1000research.73231.2 (PMC9445564; doi:10.12688/f1000research.73231.2)
Supplement: Peer review history

# Response

**Published:** 2022-09-12

**Authors:** Chee Ying Kuek

**Affiliations:** Faculty of Law, Multimedia University, Malaysia

**In response to Dr Noraiza Abdul Rahman:**

 1. The reviewer stated that abortion in Malaysia is governed under ss 312 to 316 of the Penal Code and why other sections were not mentioned. In respond to this query, the author would like to inform that the paper discussed ss 312 and 315 of the Penal Code. However, ss 313, 314 and 316 were not discussed because they are irrelevant to the current study:

 Section 313 of the Penal Code is about causing miscarriage without the woman’s consent, which is different from s 312 which deals with causing miscarriage with the consent of the pregnant woman.

 Section 314 of the Penal Code deals with instances where the woman’s death is caused by an act done with the intention to cause miscarriage.

 This is irrelevant for the current study because the current study is not about a doctor causing death to a pregnant woman while procuring an abortion.

 Section 316 of the Penal Code makes it an offence for a person to cause the death of a quick unborn child, although without intention, while committing an act that caused the death of the mother, in which he would be guilty of culpable homicide not amounting to murder.

 This is irrelevant for the current study because the current study is not about a doctor causing death to a pregnant woman.

 2. The “Discussion” section on the “Wrongful birth” has been revised by incorporating 2 studies which showed the reasons why an abortion is viewed negatively in Malaysia.

 3. The case of *ACB v Thomson Medical Pte Ltd* [2015] which dealt with a claim to recover the cost of raising a healthy baby born following a negligently performed IVF procedure was not discussed in the current study. This is because that case can be distinguished from the current study which dealt with the claim to recover the costs of the disabled child’s special needs.

# Response

**Published:** 2022-09-12

**Authors:** Chee Ying Kuek

**Affiliations:** Faculty of Law, Multimedia University, Malaysia

**In response to Associate Prof. Dr. Tengku Noor Azira bt Tengku Zainudin:**

1. A brief definition of “abortion” and “miscarriage” has been added under the section “Abortion law in Malaysia”.
2. One case law on the application of s 312 of the Penal Code, namely PP v Dr Nadason Kanagalingam (1985) has been added under the section “Abortion law in Malaysia”.
3. Under the “Discussion” section on the “Wrongful birth”, further explanation has been added to the last paragraph on circumstances that a woman may potentially bring a wrongful birth action in Malaysia.
4. Under the section “Abortion law in the United Kingdom and Singapore”, further explanation has been added on the legal position of terminating a pregnancy solely on the ground of fetus impairment in Malaysia. A distinction was made with the UK on this aspect.
5. Under the “Discussion” section on the “Wrongful birth”, further explanation has been added after the sentence “wrongful birth action is expected to be rare in Malaysia due to the cultural and religious reasons which tend to be more pro-life than pro-choice (Low, Tong, and Gunasegaran, 2013).” to support the sentence.

**In response to Dr. Mohd. Zamre bin Mohd. Zahir:**

1. The section of “Conclusion” has been expanded with further elaboration.
2. The application of s 312 of the Penal Code has been further elaborated so that its link to the current study is clearer or reinforced. The amended parts can be found under the sections “Abortion law in Malaysia”, “Abortion law in the United Kingdom and Singapore” and Discussion” section on the “Wrongful birth”.
3. The author was of the view that ss 316 and 318 of the Penal Code are irrelevant for the current study and do not need to be discussed because:

Section 316 of the Penal Code makes it an offence for a person to cause the death of a quick unborn child, although without intention, while committing an act that caused the death of the mother, in which he would be guilty of culpable homicide not amounting to murder.

 This is irrelevant for the current study because the current study is not about a doctor causing death to a pregnant woman.

 Section 318 of the Penal Code deals with concealment of birth by secret disposal of dead body. This is irrelevant to the current study because the current study discussed the legal implication of prenatal diagnosis from the aspects of, among other things, abortion that is permitted by the law (Penal Code). It is not about illegal abortion that is carried out clandestinely.

 4. The section of “Conclusion” which contained the sentence pointed out by the reviewer has been expanded with further elaboration.
